# Cryo-EM Structures of AcrD Illuminate a Mechanism for Capturing Aminoglycosides from Its Central Cavity

**DOI:** 10.1128/mbio.03383-22

**Published:** 2023-01-10

**Authors:** Zhemin Zhang, Christopher E. Morgan, Meng Cui, Edward W. Yu

**Affiliations:** a Department of Pharmacology, Case Western Reserve University School of Medicine, Cleveland, Ohio, USA; b Department of Chemistry, Thiel College, Greenville, Pennsylvania, USA; c Department of Pharmaceutical Sciences, Northeastern University School of Pharmacy, Boston, Massachusetts, USA; Dana-Farber Cancer Institute

**Keywords:** AcrD, Cryo-EM, multidrug efflux pump, resistance-nodulation-cell division, multidrug resistance

## Abstract

The Escherichia coli acriflavine resistance protein D (AcrD) is an efflux pump that belongs to the resistance-nodulation-cell division (RND) superfamily. Its primary function is to provide resistance to aminoglycoside-based drugs by actively extruding these noxious compounds out of E. coli cells. AcrD can also mediate resistance to a limited range of other amphiphilic agents, including bile acids, novobiocin, and fusidic acids. As there is no structural information available for any aminoglycoside-specific RND pump, here we describe cryo-electron microscopy (cryo-EM) structures of AcrD in the absence and presence of bound gentamicin. These structures provide new information about the RND superfamily of efflux pumps, specifically, that three negatively charged residues central to the aminoglycoside-binding site are located within the ceiling of the central cavity of the AcrD trimer. Thus, it is likely that AcrD is capable of picking up aminoglycosides via this central cavity. Through the combination of cryo-EM structural determination, mutagenesis analysis, and molecular simulation, we show that charged residues are critically important for this pump to shuttle drugs directly from the central cavity to the funnel of the AcrD trimer for extrusion.

## INTRODUCTION

Emerging multidrug resistance (MDR), particularly in Gram-negative bacteria, poses a serious threat to public health. MDR limits our effective drug library and significantly endangers our ability to treat bacterial infections. Pathogens such as Acinetobacter baumannii, Burkholderia pseudomallei, *Campylobacteria jejuni*, Klebsiella pneumoniae, Neisseria gonorrhoeae, Pseudomonas aeruginosa, and many other Gram-negative organisms are expected to become increasingly resistant and possibly untreatable within the next decade. One major mechanism these “superbugs” use to counteract the action of antimicrobials and antibiotics is the enhanced expression of multidrug efflux pumps, which contribute significantly to both intrinsic and acquired resistance to a variety of clinically relevant agents (1). Bacterial efflux pumps have enormous consequences that compromise the effectiveness of clinical therapies and lengthen the duration of treatment as they can diminish intracellular drug concentrations to the level that these pathogens can easily handle.

Among various types of efflux pumps, members of the resistance-nodulation-cell division (RND) superfamily (2) are the most critical in mediating antibiotic resistance in Gram-negative bacteria. An RND efflux pump is an inner membrane protein. It typically interacts with a periplasmic membrane fusion protein and an outer membrane channel protein to assemble as a tripartite efflux complex that spans the entire cell envelope and extrudes antimicrobials directly out of bacterial cells (3).

Escherichia coli harbors seven RND pumps that belong to the hydrophobe-amphiphile efflux (HAE) (AcrB, AcrD, AcrF, MdtB, MdtC, and YhiV) and heavy-metal efflux (HME) (CusA) subfamilies (4–12). Among them, the structures of the AcrB (13–15) HAE-RND and CusA (16–18) HME-RND have been solved. These structures have helped unravel the molecular mechanisms that govern the function of these RND pumps.

Within the HAE-RND class of membrane proteins, AcrD is distinct in that it is a specific aminoglycoside efflux pump that mediates resistance to a variety of aminoglycoside drugs, including amikacin, gentamicin, neomycin, kanamycin, and tobramycin (7). AcrD is also capable of providing resistance to a limited range of amphiphilic agents, such as bile acids, novobiocin, and fusidic acid (19). The AcrD efflux pump is a substrate/proton antiporter. It contains substrate-binding sites and utilizes the proton-motive-force (PMF) to pump these drugs out of the cell. It works in conjunction with the AcrA periplasmic membrane fusion protein and the TolC outer membrane channel to eliminate aminoglycosides and minimize the toxicity of these drugs (7, 8, 19).

Currently, there has been no structural information acquired for any aminoglycoside-specific RND pump. To elucidate the mechanisms used by AcrD for aminoglycoside recognition and extrusion, we describe cryo-EM structures of the aminoglycoside efflux pump both in the absence and presence of gentamicin (Gen). The structures suggest that AcrD relies on charged residues to bind and export these charged drugs, where the AcrD pump is capable of picking up aminoglycosides from the central cavity formed by the trimer. Our data also indicate that AcrD can capture substrates from both the cytoplasm and periplasm. Combined with site-directed mutagenesis and molecular dynamics simulation, our data provide a detailed pathway for substrate transport via the AcrD membrane protein.

## RESULTS

### Structures of AcrD in the absence of gentamicin.

We cloned full-length E. coli AcrD with a 6×His tag at the C-terminus into pSportI (19) to generate the pSportIΩ*acrD* expression vector. The AcrD protein was overproduced and purified from E. coli BL21(DE3)*ΔacrB* cells. We then reconstituted the purified AcrD pump into lipidic nanodiscs and collected single-particle images using cryo-EM (Fig. S1). Surprisingly, the cryo-EM images depict that these single particles consist of a mixture of AcrD trimers, dimers, and monomers. The particle ratio of trimer to dimer to monomer is approximately 3:5:2. Three-dimensional reconstitutions of these particles allowed us to obtain cryo-EM maps of trimeric, dimeric, and monomeric AcrD at nominal resolutions of 3.09 Å, 2.95 Å, and 7.66 Å, respectively (Fig. S1). These maps also enabled us to build models of the AcrD trimer and dimer. Each subunit of the full-length AcrD protein consists of 1,035 amino acids. Residues 1 to 1,032 are included in our final model (Table S1). As we only obtained a low resolution cryo-EM map of monomeric AcrD, we did not build a final model for this monomer.

**FIG 1:**
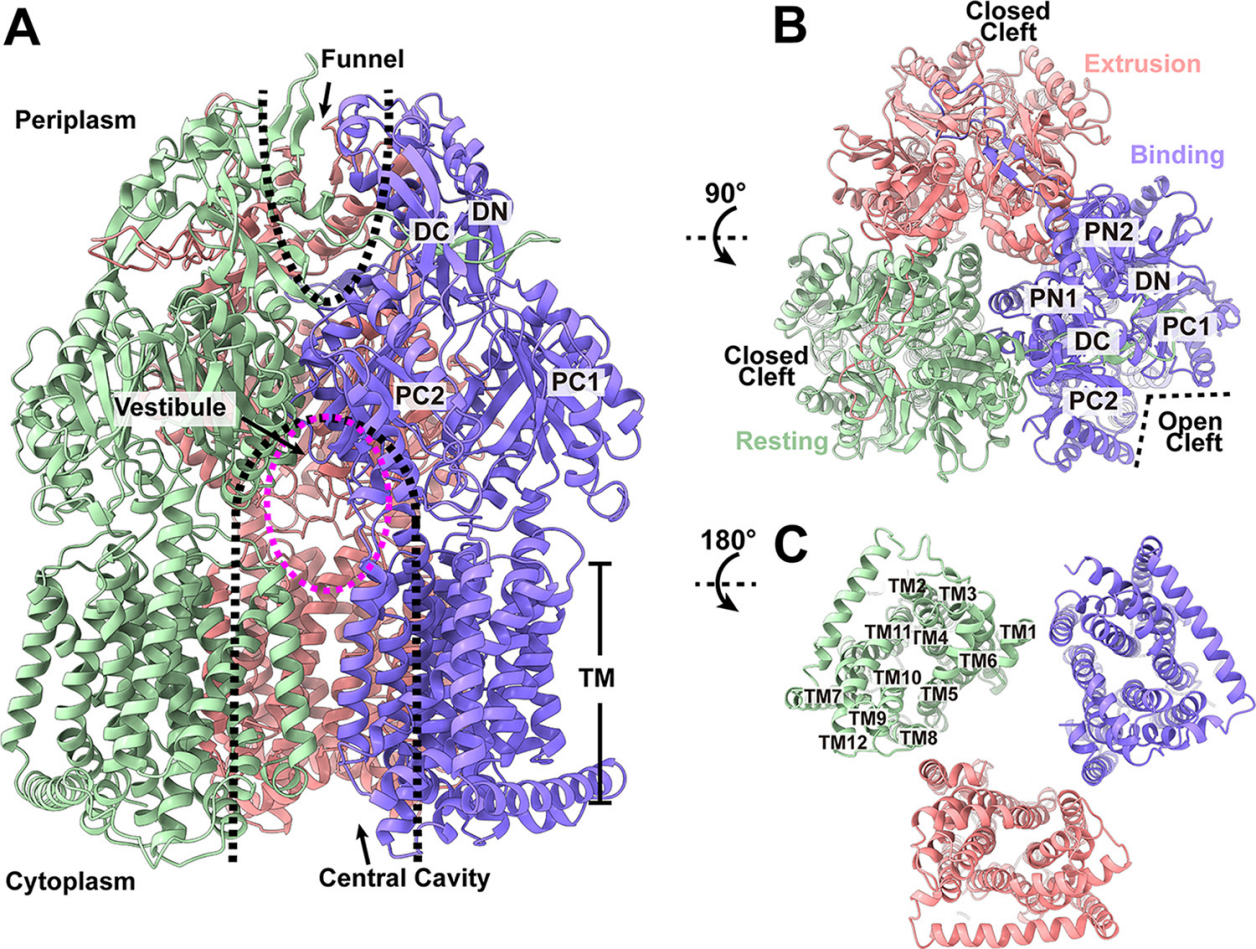
Cryo-EM structure of trimeric AcrD in the absence of Gen. Ribbon diagrams of the structure of the (A) side view (viewed in the membrane plane), (B) top view (viewed from the extracellular space), and (C) bottom view (viewed from the cytoplasm) of the AcrD trimer. In (A to C), the “binding,” “extrusion,” and “resting” protomers are colored slate, magenta, and green, respectively. Each protomer of AcrD contains 12 transmembrane helices (TM1-TM12) and six periplasmic subdomains (PN1, PN2, PC1, PC2, DN, and DC). The locations of the central cavity, vestibule and funnel are labeled.

**(i) Structure of trimeric AcrD.** The cryo-EM structure of trimeric AcrD revealed that this multidrug efflux pump adopts the overall fold of hydrophobe-amphiphile efflux (HAE)-RND-type proteins (13, 14, 20–23). Each subunit of AcrD contains a transmembrane domain and a large periplasmic domain. The transmembrane domain is composed of 12 transmembrane helices (TM1-TM12), whereas the periplasmic domain can be divided into six subdomains (PN1, PN2, PC1, PC2, DN and DC) (Fig. 1A, B, and C). This periplasmic domain is formed by two loops between TM1 and TM2 and between TM7 and TM8. In the transmembrane region, TM1-TM6 are related to TM7-TM12 by pseudo-2-fold symmetry. Similar to CusA (16–18), TM2 and TM8 extend and protrude into the periplasm and contribute to the periplasmic domain.

As expected, subdomains PC1 and PC2 create a periplasmic cleft. In E. coli AcrB (13–15), P. aeruginosa MexB (20), A. baumannii AdeB (23, 24), A. baumannii AdeJ (25, 26), C. jejuni CmeB (22), and N. gonorrhoeae MtrD (21, 27), this periplasmic cleft can be open or closed and forms an entrance to allow substrates to enter the pump from the periplasmic space.

Our cryo-EM structure of trimeric AcrD depicts that this pump forms an asymmetric trimer, featuring a conformational state of one periplasmic cleft open and two clefts closed. A detailed inspection of the structure indicates that the three protomers present three different conformations, which can be classified as “binding,” “extrusion,” and “resting” states (Fig. 1B), based on detailed structural features of these protomers (Table S2; Fig. S2). Therefore, the apo-AcrD trimer presents a very distinct conformation compared with other structures of asymmetric RND pumps found in E. coli AcrB (28), P. aeruginosa MexB (20), N. gonorrhoeae MtrD (27, 29), *A. buamannii* AdeB (24), and A. baumannii AdeJ (25, 26), where the structures of these multidrug efflux pumps showed that the three protomers present “access,” “binding,” and “extrusion” conformational states with two periplasmic clefts open and one cleft closed.

AcrD is a PMF-dependent pump that functions via an antiport mechanism. Coupled with the export of aminoglycosides, protons have to flow into the cytoplasm to energize this efflux process. Our cryo-EM maps unambiguously depict the change in conformation of residue side chains, including D407, D408, K938, N939, and T975, which form the proton-relay network within the transmembrane helices (Fig. S2).

**FIG 2:**
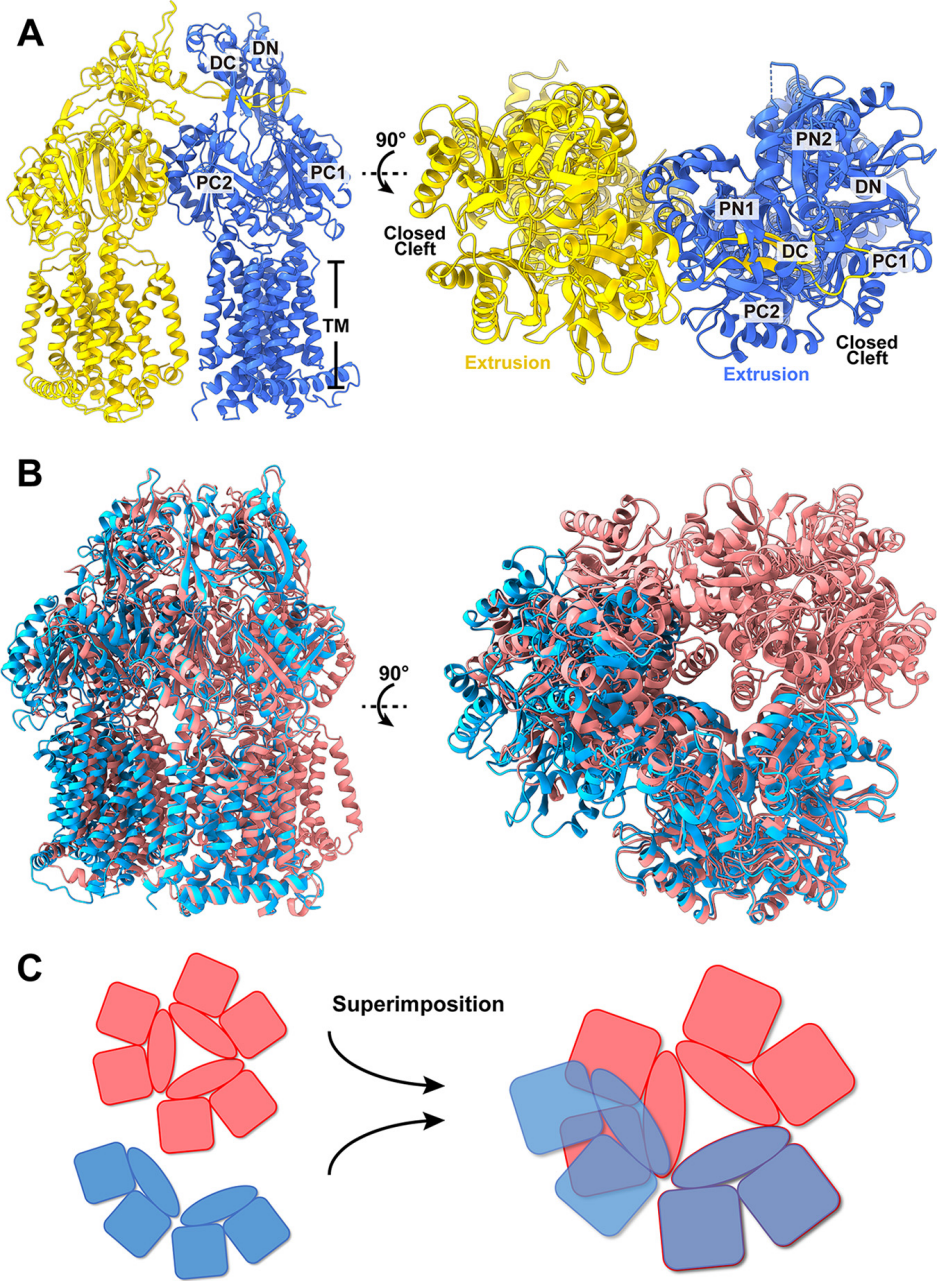
Cryo-EM structure of dimeric AcrD in the absence of Gen. Ribbon diagrams of the structure of AcrD viewed in the membrane plane (side view) and from the extracellular space (top view). The two AcrD protomers are colored yellow and blue, respectively. (B) Superimposition of the structures of trimeric and dimeric AcrD in the absence of Gen. The trimeric and dimeric AcrD protomers are colored magenta and blue, respectively. (C) A cartoon of the superimposition of trimeric and dimeric AcrD. The trimeric and dimeric AcrD protomers are colored magenta and blue, respectively.

**(ii) Structure of dimeric AcrD.** Surprisingly, our single-particle images also indicate that AcrD can assemble into a dimeric form (Fig. 2A). It is interesting to note that this dimeric oligomerization has not been previously identified among the HAE- and HME-RND families of transporters. The structure of dimeric AcrD depicts that these two AcrD protomers are nearly identical and display an “extrusion” conformational state (Fig. S2; Table S2). Superimposition of a protomer of dimeric AcrD to the “extrusion” protomer of trimeric AcrD indicates that these two protein molecules can be easily superimposed with an r.m.s.d. of 0.9 Å, suggesting that these two AcrD protomers depict a similar conformational state. In comparison with the overall structures of dimeric and trimeric AcrD, it appears that the assembly of dimeric AcrD is distinct in that the second subunit of AcrD is located at a position that is much closer to the first subunit, indicating that the dimer interface of dimeric AcrD is narrower than that of trimeric AcrD (Fig. 2B and C). Based on this unique structure, the dimeric AcrD structure may represent an intermediate that this pump must go through to progress into a trimeric assembly.

### Structures of AcrD in the presence of gentamicin.

We incubated a 10 µM AcrD-nanodisc sample with 500 µM Gen for 1 h and collected single-particle cryo-EM images of the AcrD-Gen complex. Extensive classification of the single-particle images indicated that there were three distinct populations of AcrD coexisting in the single nanodisc sample, where this pump can be trimeric, dimeric, or monomeric in oligomerization (Fig. S3). Several iterative rounds of classifications allowed us to sort the images based on these three distinct oligomerizations. The ratio of single-particle counts corresponding to these trimers, dimers, and monomers is approximately 3.4:5.6:1. Three-dimensional reconstitutions of these particles allowed us to obtain cryo-EM maps of trimeric, dimeric and monomeric AcrD in the presence of Gen at nominal resolutions of 3.06 Å, 2.98 Å, and 6.81 Å, respectively (Fig. S3). These maps also enabled us to build models of the AcrD trimer and dimer (Table S1). However, we did not build a final structural model of monomeric AcrD as the resolution of this cryo-EM map is quite low.

Residues 1 to 1,032 are included in the final model of each protomer of the trimeric and dimeric AcrD structures. Surprisingly, we observed that only the trimeric oligomerization of AcrD contains a Gen molecule, whereas the dimeric AcrD pump is absent of bound ligand.

**FIG 3:**
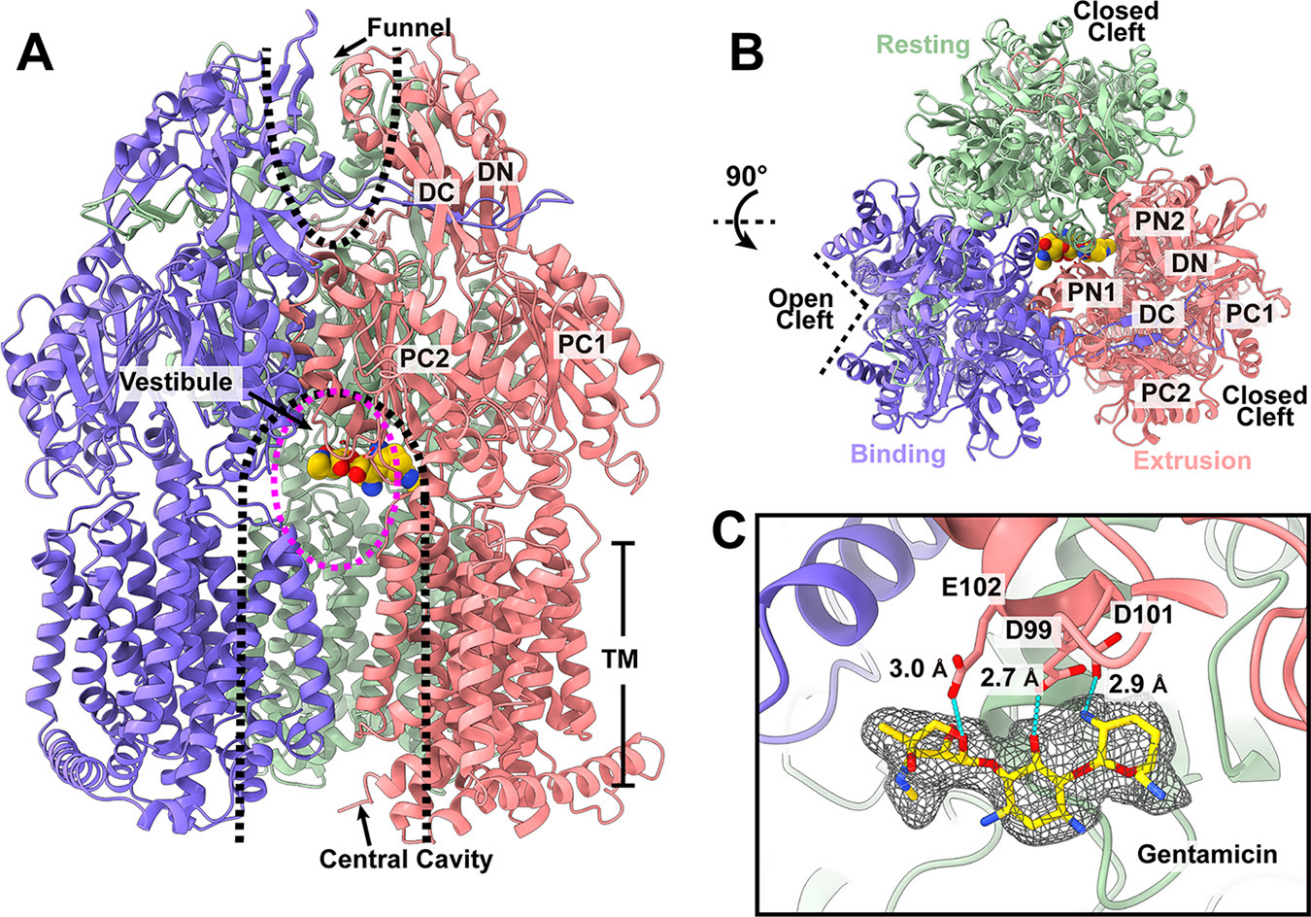
Cryo-EM structure of trimeric AcrD in the presence of Gen. (A) Ribbon diagram of the structure of the side view (viewed in the membrane plane) of the AcrD trimer. The locations of the central cavity, vestibule and funnel are highlighted with dotted lines. (B) Ribbon diagram of the structure of the top view (viewed from the extracellular space) of the AcrD trimer. In (A) and (B), the “binding,” “extrusion,” and “resting” protomers are colored slate, magenta, and green, respectively. Each protomer of AcrD contains 12 transmembrane helices (TM1-TM12) and six periplasmic subdomains (PN1, PN2, PC1, PC2, DN, and DC). The bound Gen drug is shown as yellow balls. (C) The Gen-binding site. Residues D99, D101, and E102 individually form hydrogen bonds (cyan dotted lines) with bound Gen. The bound Gen molecule is in yellow sticks. Cryo-EM densities of bound Gen are in black meshes.

**(i) Structure of trimeric AcrD in the presence of Gen.** The structure of trimeric AcrD bound with Gen (AcrD-Gen) (Fig. 3A) is very similar to that of trimeric AcrD in its unbound form (apo-AcrD), as they both present as asymmetric trimers. Superimposition of these two trimers give rise to an r.m.s.d. of 0.4 Å, suggesting that the structures of these two trimers are very similar to each other (Fig. S4). As with the apo form of the AcrD trimer, the AcrD-Gen structure depicts that one of the AcrD protomers prefers a transient state with its periplasmic cleft open, whereas the periplasmic clefts of other two protomers remain closed (Fig. 3B). A closer inspection of the trimeric structure indicates that the trimer contains a “binding” protomer (open periplasmic cleft), an “extrusion” protomer (closed periplasmic cleft) and a “resting” protomer (closed periplasmic cleft) (Fig. 3B; Fig. S2).

Unexpectedly, an extra density was found at the ceiling of the central cavity formed by the AcrD trimer (Fig. 3C). The shape of this density is compatible with a Gen molecule, indicating that Gen is bound by AcrD at this location. It was observed that the “extrusion” protomer of AcrD uses D99, D101, and E102 to anchor this drug (Fig. 3C). These three negatively charged residues participate in forming hydrogen bonds with Gen to secure the binding.

**FIG 4:**
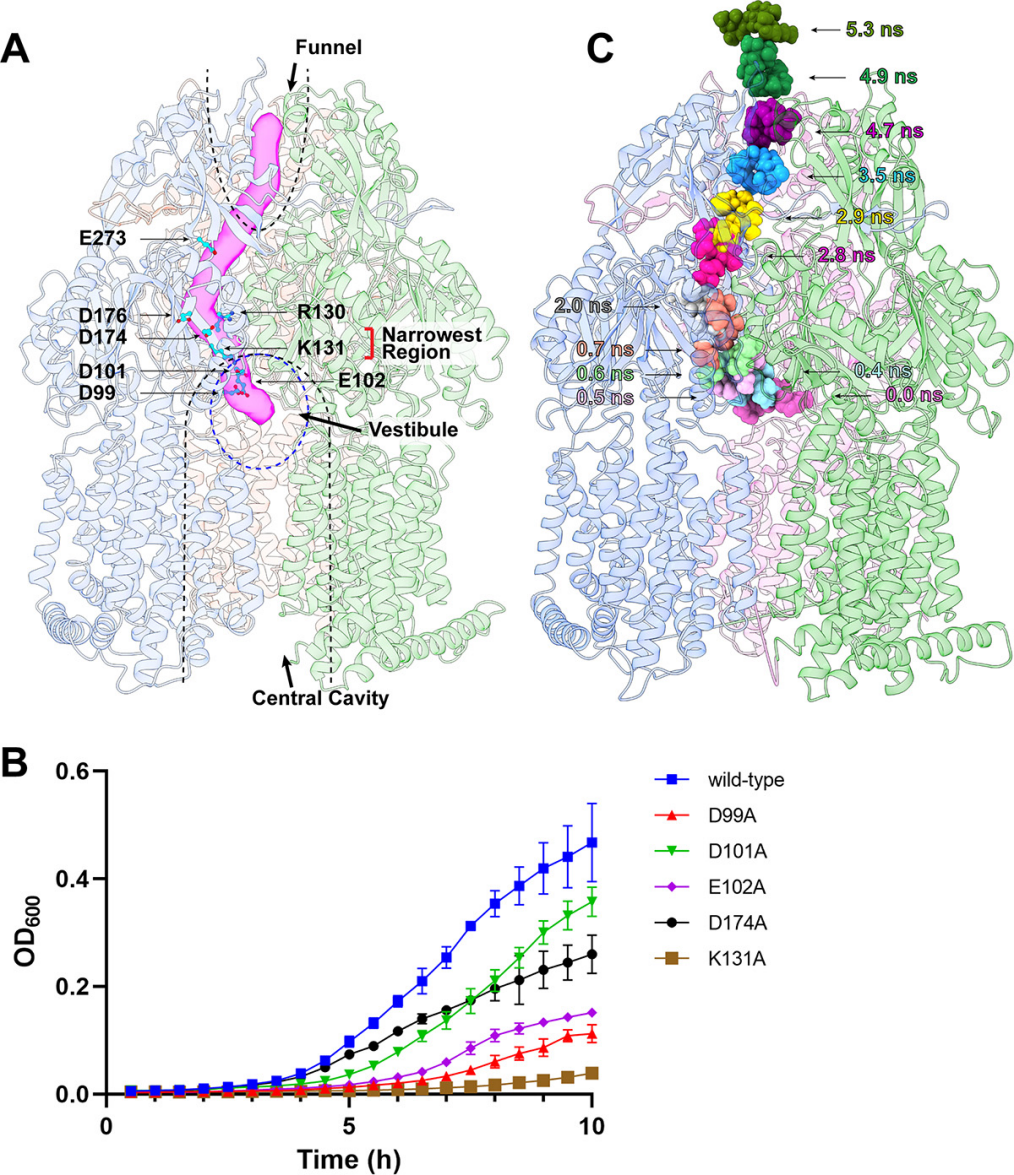
Mutagenesis and target MD simulation of AcrD efflux pump. (A) The “extrusion” protomer forms a periplasmic tunnel (colored pink) important for drug extrusion. Several charged residues, including R130, K131, D174, D176, and E273, are found to line the wall of the periplasmic tunnel. Residues K131 and D174 form the narrowest region of this tunnel. Residues D99, D101, and E102, which create the Gen-binding site, are also included. The “binding,” “extrusion,” and “resting” protomers are colored slate, magenta, and green, respectively. The locations of the central cavity, vestibule and funnel are highlighted with dotted lines. (B) Growth of E. coli JW2454-1Δ*acrD*/pSportIΩ*acrD* cells in the presence of 6 µg/mL Gen. Growth of cells harboring the mutants D99A, D101A, E102A, K131A, and D174A are inhibited by 75.9% (*P* < 0.0001; Student's *t* test), 23.5% (*P* < 0.01; Student's *t* test), 67.7% (*P* < 0.0001; Student's *t* test), 91.7% (*P* < 0.0001; Student's *t* test), and 44.4% (*P* < 0.0001; Student's *t* test) compared with cells carrying the wild-type AcrD efflux pump at the 10 h time point. (C) Target MD simulations of the AcrD pump. The calculations depict snapshots (0, 0.4, 0.5, 0.6, 0.7, 2.0, 2.8, 2.9, 3.5, 4.7, 4.9, and 5.3 ns) of Gen shuttling via the periplasmic tunnel of AcrD.

A tunnel is formed at the periplasmic domain of the “extrusion” protomer, which binds Gen at the central cavity via hydrogen bonds. This tunnel spans the entire periplasmic region of the “extrusion” protomer, starting from the drug binding site at the ceiling of the central cavity to the bottom of the periplasmic funnel created by subdomains DN and DC (Fig. 4A). This tunnel includes the negatively charged triad formed by D99, D101, and E102 as well as the charged residues R130, K131, D174, and D176. It is interesting to note that residues K131 and D174 create the narrowest region of this periplasmic tunnel (Fig. 4A).

**(ii) Structure of dimeric AcrD in the presence of Gen.** The structure of dimeric AcrD in the presence of Gen is essentially identical to that of dimeric AcrD in the absence of Gen. Superimposition of these two dimeric structures gives rise to an r.m.s.d. of 0.2 Å (Fig. S4). Like dimeric apo-AcrD, the two protomers of AcrD both present an “extrusion” conformational state of this pump (Fig. S2).

### Mutagenesis of charged residues involved in forming the periplasmic tunnel.

The structure of AcrD-Gen highlights the importance of charged residues lining the periplasmic tunnel that connects the central cavity and funnel of the AcrD trimer. These residues create the aminoglycoside-binding site (residues D99, D101, and E102) and a charged network (residues R130, K131, D174, and D176) to line the wall of the periplasmic tunnel. These charged amino acids are likely critical for the transport of aminoglycosides out of bacterial cells. To test this, we decided to study residues forming the drug-binding site (D99, D101, and E102) and the narrowest region of the tunnel (K131 and D174) using site-directed mutagenesis. We made single-point mutants (D99A, D101A, E102A, K131A and D174A) of these charged residues. Each individual point mutation of AcrD was separately expressed in E. coli JW2454-1*ΔacrD* cells lacking the *acrD* gene. The expression level of each mutant was similar to that of the wild-type transporter as indicated by Western analysis (Fig. S5).

We then measured the growth of these cells in the presence of 6 µg/mL Gen (the MIC for Gen was found to be 8 µg/mL). After 10 h, growth levels of cells expressing D99A, D101A, E102A, K131A, or D174A AcrD were reduced by 75.9% (*P* < 0.0001; Student's *t* test), 23.5% (*P* < 0.01; Student's *t* test), 67.7% (*P* < 0.0001; Student's *t* test), 91.7% (*P* < 0.0001; Student's *t* test) and 44.4% (*P* < 0.0001; Student's *t* test) in comparison with the growth of E. coli JW2454-1*ΔacrD* cells carrying wild-type AcrD (Fig. 4B). The results strongly suggest that these charged residues are important for the function of this efflux pump.

### Computational simulation of the AcrD aminoglycoside efflux pump.

To elucidate the mechanism of aminoglycoside transport, we performed molecular dynamics (MD) and target MD simulations on the AcrD-Gen complex in an explicit lipid bilayer and water environment using Amber (30, 31) and NAMD (32) (Fig. S6). We observed that the Gen molecule indeed follows the path of the tunnel identified from the cryo-EM structure of AcrD-Gen to shuttle across the periplasmic domain of AcrD (Fig. 4C). Based on target MD simulations, Gen first enters the AcrD pump via the central cavity binding site, which includes residues D99, D101, and E102. It then passes through the tunnel, interacting with several charged residues, including R130, K131, D174, D176, and E273, and eventually reaches the funnel region and leaves this membrane protein through the exit site formed by Q125 and Y756 (Fig. S2). Our data show that Gen can arrive at the funnel within 4 ns (Fig. 4C). It is worth mentioning that we previously used target MD simulation to study the drug transport process in the A. baumannii AdeB multidrug efflux pump (24). The calculated result showed that ~15 ns is required for a drug molecule to transport from the entrance drug-binding site of the periplasmic cleft to the funnel region. Our simulation in this study indeed indicates that the drug transport process via the central cavity drug-binding site is quite efficient.

## DISCUSSION

We have defined cryo-EM structures of the AcrD aminoglycoside efflux pump in both the absence and presence of Gen. These structural data allow us to observe new conformational and oligomerization states that have not been previously identified for RND multidrug efflux pumps. Surprisingly, our single-particle images indicate that the oligomerization state of AcrD can be monomeric and dimeric, in addition to the typical trimeric form of this class of membrane proteins. We used the identical composition of nanodiscs, identical protein purification procedures, and similar E. coli protein expression systems to produce cryo-EM samples for structural determinations of the A. baumannii AdeB (23, 24), A. baumannii AdeJ (25, 26), and N. gonorrhoeae MtrD (27, 29) multidrug efflux pumps. We could only detect the trimeric oligomerization of these pumps. Therefore, it is not likely that the observed mixture of monomeric, dimeric, and trimeric AcrD in the sample is due to the preparation and composition of nanodiscs. These images may represent snapshots of different intermediate states toward the process for trimeric oligomerization of this pump (Fig. S7).

Several years ago, we pursued crystallographic study of the AcrD pump. However, the AcrD crystals poorly performed in the X-ray beam and the best AcrD crystal only diffracted X-rays to ~10 Å. The existence of different oligomerization states of this purified membrane protein helps to explain the poor quality of these crystals.

In both the absence and presence of Gen, we found substantial particles of the dimeric and trimeric forms, which allowed us to solve structures of these oligomerization states of AcrD at near atomic resolutions. Our results suggest that AcrD in both apo and Gen bound forms assemble as asymmetric trimers. Interestingly, the conformational states of these trimers are quite distinct from those found in the structures of E. coli AcrB (14), N. gonorrhoeae MtrD (27), C. jejuni CmeB (22), A. baumannii AdeB (24), and A. baumannii AdeJ (25, 26). It appears that these trimeric HAE-RND pumps are also able to form symmetric trimers as seen in cases of E. coli AcrB (13, 15, 33), N. gonorrhoeae MtrD (21), and C. jejuni CmeB (22), as well as the HME-RND pump E. coli CusA (16). Previously, we investigated the efflux mechanisms of C. jejuni CmeB (22) and E. coli CusA (34) using single-molecule fluorescence resonance energy transfer (single-molecule FRET) and single-particle cryo-EM, respectively. We observed that each protomer of these two trimeric efflux pumps undergoes conformational transitions uncoordinated and independent of each other. Thus, the unique conformation of our structures of trimeric AcrD in relation to structures of other RND pumps may also indicate that the conformational state of individual AcrD protomers may not be correlated with each other within the trimer.

Our AcrD structures provide strong evidence that this pump can pick up aminoglycosides from the central cavity of the AcrD trimer. This result may imply that AcrD is capable of extruding drugs from both the periplasm and cytoplasm. Indeed, it has been previously demonstrated that aminoglycosides can be captured from the periplasm and cytoplasm by AcrD (8). This dual-pathway phenomenon for the removal of toxic compounds from bacterial cells has been corroborated in the CusA efflux pump, where CusA is capable of transporting Cu(I)/Ag(I) from the cytosol in addition to capturing these toxic ions from the periplasmic space (16). Thus, we believe that AcrD can pick up the aminoglycosides from both the cytoplasm and periplasm and uses charged residues to bind and export these charged drugs.

Similar to AcrB, the AcrD pump should be able to uptake drugs from the periplasmic cleft created between subdomains PC1 and PC2. This cleft can be open or closed and form the entrance drug-binding site that marks the first step of drug transport via the periplasmic space. An aminoglycoside molecule recognized by the entrance drug-binding site will be guided by the F-loop to arrive at the proximal drug-binding site. Subsequently, this sugar-based drug will pass through the G-loop and reach the distal drug-binding site for extrusion (Fig. S8).

**FIG 5:**
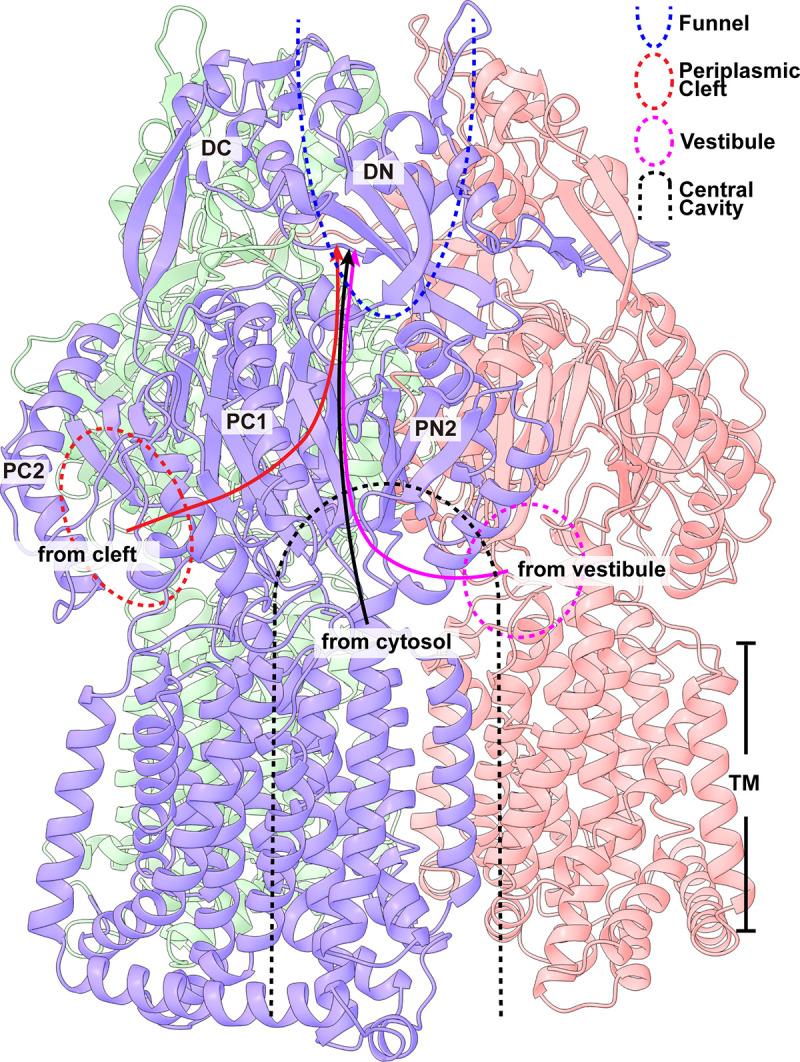
The three pathways for drug extrusion in AcrD. The three solid curves represent three possible drug extrusion pathways (red, from cleft; magenta, from vestibule; black, from cytosol). The locations of the periplasmic cleft, vestibule, central cavity, and funnel are highlighted with red, magenta, black, and blue colors, respectively.

In addition to the periplasmic cleft, it has been shown that the AcrB multidrug efflux pump is able to capture drug molecules from the vestibule (35) that forms an opening at the membrane surface and between the interface of two protomers (Fig. 5). Deep inside this vestibule is the ceiling of the central cavity, where the negatively charged triad formed by residues D99, D101, and E102 is located. Our cryo-EM work showed that this charged triad creates an aminoglycoside binding site in the central cavity. A similar position of this binding site at the central cavity of a trimeric multidrug efflux pump has been previously demonstrated in the AcrB membrane protein using X-ray crystallography (15, 33). This central cavity binding site was also confirmed by another research group by cocrystallizing AcrB with linezolid (36). Further, site-directed mutagenesis of the Pseudomonas fluorescens EmhB multidrug efflux pump, a homolog of AcrB and AcrD, indicated that residues at the top portion of the central cavity are important for drug efflux (37). Based on our structural data, we therefore believe that the AcrD vestibule may form a possible pathway for this pump to capture aminoglycosides.

In addition to these two periplasmic pathways, the AcrD pump may catch drugs from the cytoplasm via the central cavity of the trimer which spans the entire inner membrane of E. coli and leads to the cytosol (Fig. 5). Thus, an aminoglycoside drug may enter the central cavity via the vestibule and/or cytoplasm and bind at the ceiling of the central cavity. This bound drug molecule anchored by the three negatively charged binding site formed by D99, D101, and E102 could then pass through the tunnel lining with charged residues, including R130, K131, D174, and D176, for drug export. Therefore, transport of an aminoglycoside molecule via either the periplasm or cytoplasm is likely to involve a stepwise process that shuttles the drug through these charged residues, after which it could be released to the central funnel of the AcrD trimer for final drug extrusion.

## MATERIALS AND METHODS

### Expression and purification of AcrD.

The E. coli AcrD aminoglycoside efflux pump was cloned into the pSportIΩ*acrD* expression vector in frame with a 6×His tag at the C-terminus. The plasmid was transfected into E. coli BL21(DE3)*ΔacrB* cells, which harbor a deletion in the chromosomal *acrB* gene, for overproduction of the AcrD membrane protein. Cells were grown in 6 L of Luria-Bertani (LB) medium supplemented with 100 µg/mL ampicillin at 37°C. When the OD_600 nm_ reached 0.5, the expression of AcrD was induced with 0.2 mM isopropyl-β-d-thiogalactopyranoside (IPTG). Cells were then harvested within 4 h of induction. The collected bacterial cells were resuspended in low salt buffer (100 mM sodium phosphate [pH 7.2], 10% glycerol, 1 mM ethylenediaminetetraacetic acid [EDTA], and 1 mM phenylmethanesulfonyl fluoride [PMSF]) and disrupted with a French pressure cell. The membrane fraction was collected and washed twice with high salt buffer (20 mM sodium phosphate [pH 7.2], 2 M KCl, 10% glycerol, 1 mM EDTA and 1 mM PMSF), and once with final buffer (20 mM HEPES-NaOH buffer [pH 7.5] and 1 mM PMSF). The membrane protein was then solubilized in 2% (wt/vol) n-dodecyl-β-d-maltoside (DDM). Insoluble material was removed by ultracentrifugation at 100,000 × *g*. The extracted protein was then purified with a Ni^2+^-affinity column. The purity of the AcrD protein (>95%) was judged using SDS-PAGE stained with Coomassie brilliant blue. The purified protein was dialyzed against 20 mM Na-HEPES (pH 7.5) and concentrated to 7 mg/mL (60 μM) in a buffer containing 20 mM Na-HEPES (pH 7.5) and 0.05% DDM.

### Nanodisc preparation.

To assemble AcrD into nanodiscs, a mixture containing 20 µM AcrD, 45 µM membrane scaffold protein 1E3D1 (MSP1E3D1) (Sigma-Aldrich) and 930 µM E. coli total extract lipid was incubated at room temperature for 15 min. 0.8 mg/mL prewashed Bio-beads (Bio-Rad) was added to remove the DDM detergent. The resultant mixture was incubated for 1 h on ice followed by overnight incubation at 4°C. The protein-nanodisc solution was filtered through 0.22 µm nitrocellulose-filter tubes to remove the Bio-beads. To separate free nanodiscs from AcrD-loaded nanodiscs, the filtered protein-nanodisc solution was purified using a Superose 6 column (GE Healthcare) equilibrated with 20 mM Tris-HCl, pH 7.5, and 100 mM NaCl. Fractions corresponding to the size of the trimeric AcrD-nanodisc complex were collected for cryo-EM sample preparation.

### Cryo-EM sample preparation.

For imaging AcrD in the absence of Gen, a 10 µM AcrD-nanodisc sample was directly applied to glow-discharged holey carbon grids (Quantifoil Cu R1.2/1.3, 300 mesh), blotted for 18 s and then plunge-frozen in liquid ethane using a Vitrobot (Thermo Fisher Scientific). For imaging AcrD in the presence of Gen, a 10 µM AcrD-nanodisc sample was incubated with 500 µM Gen for 1 h to form the AcrD-Gen complex. The sample was then applied to glow-discharged holey carbon grids (Quantifoil Cu R1.2/1.3, 300 mesh), blotted for 18 s and then plunge-frozen in liquid ethane using a Vitrobot (Thermo Fisher Scientific). All grids were then transferred into cartridges prior to data collection.

### Data collection.

For the AcrD sample in the absence of Gen, the images were collected in superresolution mode at 81 K magnification on a Titan Krios equipped with a K3 direct electron detector (Gatan). The physical pixel size was 1.07 Å/pix (superresolution of 0.535 Å/pix). Each micrograph was exposed to a total dose of 35 e-/Å^2^ for 3.5 s and 39 frames were captured using SerialEM (38). For the AcrD sample incubated with Gen, each micrograph was collected over 38 frames with a total dose of 35 e-/Å^2^ over 3.5 s using SerialEM (38).

### Data processing.

For AcrD in the absence of Gen, the superresolution image stack was aligned and binned by 2 using patch motion. The contrast transfer function (CTF) was estimated using patch CTF in cryoSPARC (39). A procedure for blob picker followed by 2D classification were applied to generate templates for automated template picking. Initially, 7,192,708 particles were selected after autopicking in cryoSPARC (39). Several iterative rounds of 2D classifications followed by *ab initio* and heterogeneous three-dimensional (3D) classifications were performed to remove false picks and classes with unclear features, ice contamination or carbon. The 3D classification analysis was then employed, resulting in three distinct classes of AcrD images. A single round of nonuniform refinement followed by local refinement with nonuniform sampling resulted in 3.09 Å, 2.95 Å, and 7.66 Å resolution cryo-EM maps for trimeric, dimeric and monomeric AcrD based on the gold standard Fourier shell correlation (FSC 0.143) (Fig. S1).

For AcrD in the presence of Gen, the same procedure was used to generate templates for automated template picking. Initially, 4,080,301 particles were selected after autopicking in cryoSPARC (39). Several iterative rounds of 2D classifications, *ab initio* and heterogeneous 3D classifications were performed to remove false picks and classes with unclear features. The 3D classification analysis also resulted in three distinct classes of AcrD images. Nonuniform refinement followed by local refinement with nonuniform sampling resulted in 3.06 Å, 2.98 Å, and 6.81 Å resolution cryo-EM maps for trimeric, dimeric and monomeric AcrD in the presence of Gen based on the gold standard Fourier shell correlation (FSC 0.143) (Fig. S3).

### Model building and refinement.

Model buildings of trimeric and dimeric AcrD were based on the cryo-EM maps, respectively. A predicted AcrD structure using AlphaFold (40) was used and fitted into the corresponding density maps using Chimera (41). The subsequent model rebuilding was performed using Coot (42). Structural refinements were performed using the phenix.real_space_refine program (28) from the PHENIX suite (43). The final atomic model was evaluated using MolProbity (44). The statistics associated with data collection, 3D reconstruction and model refinement are included in Table S1.

### Growth of E. coli cells.

E. coli JW2454-1Δ*acrD* cells, which harbor the pSportI plasmid expressing the wild-type or mutant AcrD protein, were grown in LB medium containing 100 µg/mL ampicillin at 37°C to an OD_600_ of 0.4. The bacterial cell cultures were diluted by 100 times with LB supplemented with 100 µg/mL ampicillin, 6 µg/mL gentamicin, and 0.1 mM IPTG. Cells were then inoculated in a 96-well plate with a total volume of 100 μL in each well. The experiments were performed at 37°C on a microplate reader (BioTek). The OD_600_ value was monitored and recorded every 30 min within a 24 h period. Each experiment was repeated a minimum of three times.

### MD simulations.

The protonation states of the titratable residues of the AcrD pump were determined using the H++ server (http://biophysics.cs.vt.edu/). The trimeric AcrD-Gen structure was immersed in an explicit lipid bilayer of POPC and POPE with molecular ratio of 1:1, and a water box with dimensions of 143.9 Å × 141.9 Å × 177.9 Å using the CHARMM-GUI Membrane Builder webserver (http://www.charmm-gui.org/?doc=input/membrane). Then, 150 mM NaCl and extra neutralizing counter ions were added into the system. The total number of atoms was 283,658. The Antechamber module of AmberTools was employed to generate parameters for Gen using the general AMBER force field (GAFF) (30, 31). The partial charges of Gen were calculated using *ab initio* quantum chemistry at the HF/6-31G* level (GAUSSIAN 16 program) (Gaussian Inc., Wallingford). The tleap program, utilizing ff14SB and Lipid17 force fields for protein and lipids, respectively, was used to generate parameter and coordinate files. The PMEMD.CUDA program implemented in AMBER18 (AMBER 2018, UCSF) was used to conduct MD simulations. The simulations were performed with periodic boundary conditions to produce isothermal-isobaric ensembles. Long range electrostatics were calculated using the particle mesh Ewald (PME) method (45) with a 10 Å cutoff. Prior to the calculations, energy minimization of these systems was carried out. Subsequently, the systems were heated from 0 K to 303 K using Langevin dynamics with the collision frequency of 1 ps^−1^. During heating, the AcrD pump was position-restrained using an initial constant force of 500 kcal/mol/Å^2^ and weakened to 10 kcal/mol/Å^2^, allowing lipid and water molecules to move freely. Then, the systems went through 5 ns equilibrium MD simulations. Finally, a total of 1 µs production MD simulations were conducted. During simulations, the coordinates were saved every 500 ps for analysis. The system was well equilibrated after 100 ns simulations according to root mean square deviations (RMSDs) of the protein Cα atoms (Fig. S6). GROMCAS analysis tools were used for the MD simulation trajectory analysis (46).

### Target MD simulations.

Target MD (TMD) was performed using the NAMD program (32) with the same AMBER force field parameters as described above. Gen was docked into the trimic AcrD structure using the Glide program (Schrödinger LLC). In the simulations, we selected the heavy atoms of the Gen ligand bound at the Gen binding site to be guided toward the target position of the ligand by the application of steering forces. The root mean square (RMS) distance between the current coordinates and the target structure was calculated at each timestep. The force on each selected atom was given by a gradient of potential as a function of the RMS values. Then, TMD simulation was performed for 6 ns based on the MD equilibrated coordinates. A value of 500 kcal/mol/Å^2^ was used as an elastic constant for TMD forces during the simulations.

### Data availability.

Atomic coordinates and EM maps for trimeric AcrD and dimeric AcrD in the absence of gentamicin have been deposited with PDB accession codes 8F3E and 8F4N, and EMDB accession codes EMD-28848 and EMD-28855. Atomic coordinates and EM maps for trimeric AcrD and dimeric AcrD in the presence of gentamicin have also been deposited with PDB accession codes 8F4R and 8F56, and EMDB accession codes EMD-28857 and EMD-28861.

10.1128/mbio.03383-22.1 FIG S1 Cryo-EM structure of the AcrD efflux pump in the absence of Gen. (A) Data processing workflow of trimeric, dimeric, and monomeric AcrD. Side and top views of the cryo-EM density maps of trimeric, dimeric, and monomeric AcrD are shown. (B, C, D) Representative 2D classes of trimeric, dimeric, and monomeric AcrD. (E, F, G) Gold-Standard Fourier shell correlation (GS-FSC) curves of trimeric, dimeric, and monomeric AcrD. (H, I) Local cryo-EM density maps of trimeric and dimeric AcrD. Download FIG S1, JPG file, 1.3 MB. Copyright © 2023 Zhang et al. 2023 Zhang et al. https://creativecommons.org/licenses/by/4.0/ This content is distributed under the terms of the Creative Commons Attribution 4.0 International license.

10.1128/mbio.03383-22.2 FIG S2 Measurements of the exit sites and proton-relay networks. (A) Protomers of AcrD in the absence of Gen. (a) Ribbon diagram of an AcrD protomer of dimeric AcrD showing the locations of the exit site (red box) and proton-relay network (black box). (b) Distance between the Cα atoms of Q125 and Y756, which form the exit site for drug, for the two AcrD protomers in the dimeric AcrD structure. (c) Conformational states of the proton-relay network of the two AcrD protomers in the dimeric AcrD structure. (d) Distance between the Cα atoms of Q125 and Y756, which form the exit site for drug, for the three AcrD protomers in the trimeric AcrD structure. (e) Conformational states of the proton-relay network of the three AcrD protomers in the trimeric AcrD structure. (B) Protomers of AcrD in the presence of Gen. (a) Ribbon diagram of an AcrD protomer of dimeric AcrD showing the locations of the exit site (red box) and proton-relay network (black box). (b) Distance between the Cα atoms of Q125 and Y756, which form the exit site for drug, for the two AcrD protomers in the dimeric AcrD structure. (c) Conformational states of the proton-relay network of the two AcrD protomers in the dimeric AcrD structure. (d) Distance between the Cα atoms of Q125 and Y756, which form the exit site for drug, for the three AcrD protomers in the trimeric AcrD structure. (e) Conformational states of the proton-relay network of the three AcrD protomers in the trimeric AcrD structure. Download FIG S2, JPG file, 1.6 MB. Copyright © 2023 Zhang et al. 2023 Zhang et al. https://creativecommons.org/licenses/by/4.0/ This content is distributed under the terms of the Creative Commons Attribution 4.0 International license.

10.1128/mbio.03383-22.3 FIG S3 Cryo-EM structure of the AcrD efflux pump in the presence of Gen. (A) Data processing workflow of trimeric, dimeric and monomeric AcrD. Side and top views of the cryo-EM density maps of trimeric, dimeric and monomeric AcrD are shown. (B, C, D) Representative 2D classes of trimeric, dimeric and monomeric AcrD. (E, F, G) Gold-Standard Fourier shell correlation (GS-FSC) curves of trimeric, dimeric and monomeric AcrD. (H, I) Local cryo-EM density maps of trimeric and dimeric AcrD. Download FIG S3, JPG file, 1.3 MB. Copyright © 2023 Zhang et al. 2023 Zhang et al. https://creativecommons.org/licenses/by/4.0/ This content is distributed under the terms of the Creative Commons Attribution 4.0 International license.

10.1128/mbio.03383-22.4 FIG S4 Superimposition of the structures of dimeric AcrD in the absence and presence of Gen. (A) Side view of the superimposition. (B) Top view of the superimposition. In (A) and (B), the structures of dimeric AcrD in the absence and presence of Gen are colored blue and yellow, respectively. Download FIG S4, JPG file, 2.2 MB. Copyright © 2023 Zhang et al. 2023 Zhang et al. https://creativecommons.org/licenses/by/4.0/ This content is distributed under the terms of the Creative Commons Attribution 4.0 International license.

10.1128/mbio.03383-22.5 FIG S5 Expression level of the AcrD transporters. This is an immunoblot against AcrD of membrane extracts from 50 µg dry cells of strain E. coli JW2454-1*ΔacrD* expressing the AcrD transporters (from right to left: marker, purified AcrD, empty vector, D174A, K131A, E102A, D101A, D99A, wild-type AcrD). Download FIG S5, JPG file, 0.6 MB. Copyright © 2023 Zhang et al. 2023 Zhang et al. https://creativecommons.org/licenses/by/4.0/ This content is distributed under the terms of the Creative Commons Attribution 4.0 International license.

10.1128/mbio.03383-22.6 FIG S6 MD simulation of the AcrD trimer. This is a 1 µs simulation. The RMSDs (root mean square deviations) of Cα atoms are based on the MD simulation trajectories. Download FIG S6, JPG file, 0.2 MB. Copyright © 2023 Zhang et al. 2023 Zhang et al. https://creativecommons.org/licenses/by/4.0/ This content is distributed under the terms of the Creative Commons Attribution 4.0 International license.

10.1128/mbio.03383-22.7 FIG S7 A schematic diagram showing a stepwise process for forming the AcrD trimer. (A) Top view of the three AcrD protomers. (B) Side view of the three AcrD protomers. This cartoon indicates that the process for trimeric oligomerization involves dimerization of AcrD as an intermediate step. In (A) and (B) the three AcrD protomers are colored green, blue, and red. Download FIG S7, JPG file, 0.7 MB. Copyright © 2023 Zhang et al. 2023 Zhang et al. https://creativecommons.org/licenses/by/4.0/ This content is distributed under the terms of the Creative Commons Attribution 4.0 International license.

10.1128/mbio.03383-22.8 FIG S8 Periplasmic cleft of AcrD. (A) Entrance drug-binding site. (B) Proximal drug-binding site. (C) Distal drug-binding site. In A to C, residues that are predicted to be important for drug binding are shown as cyan sticks. The F-loop and G-loop are colored yellow and orange, respectively. Download FIG S8, JPG file, 1.1 MB. Copyright © 2023 Zhang et al. 2023 Zhang et al. https://creativecommons.org/licenses/by/4.0/ This content is distributed under the terms of the Creative Commons Attribution 4.0 International license.

10.1128/mbio.03383-22.9 TABLE S1 AcrD cryo-EM data collection and refinement statistics. Download Table S1, PDF file, 0.1 MB. Copyright © 2023 Zhang et al. 2023 Zhang et al. https://creativecommons.org/licenses/by/4.0/ This content is distributed under the terms of the Creative Commons Attribution 4.0 International license.

10.1128/mbio.03383-22.10 TABLE S2 State assignment of AcrD protomer. Download Table S2, PDF file, 0.1 MB. Copyright © 2023 Zhang et al. 2023 Zhang et al. https://creativecommons.org/licenses/by/4.0/ This content is distributed under the terms of the Creative Commons Attribution 4.0 International license.
