# Supplementary figures and images for: Cryo-EM Structures of AcrD Illuminate a Mechanism for Capturing Aminoglycosides from Its Central Cavity
Source: mBio. 2023 Jan 10;14(1):e03383-22. doi: 10.1128/mbio.03383-22 (PMC9973356; doi:10.1128/mbio.03383-22)

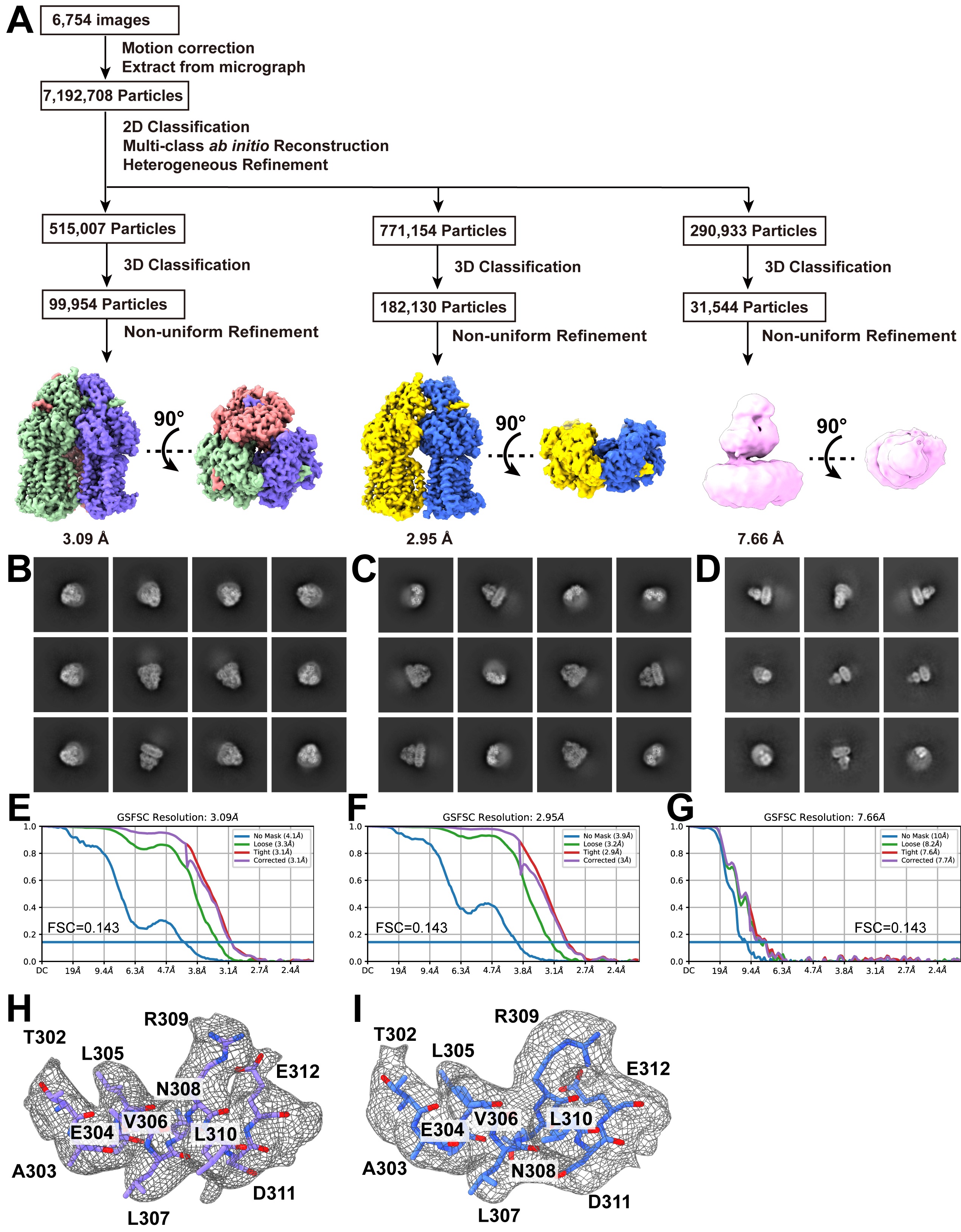

Supplement: FIG S1 [file mbio.03383-22-s0001.jpg]

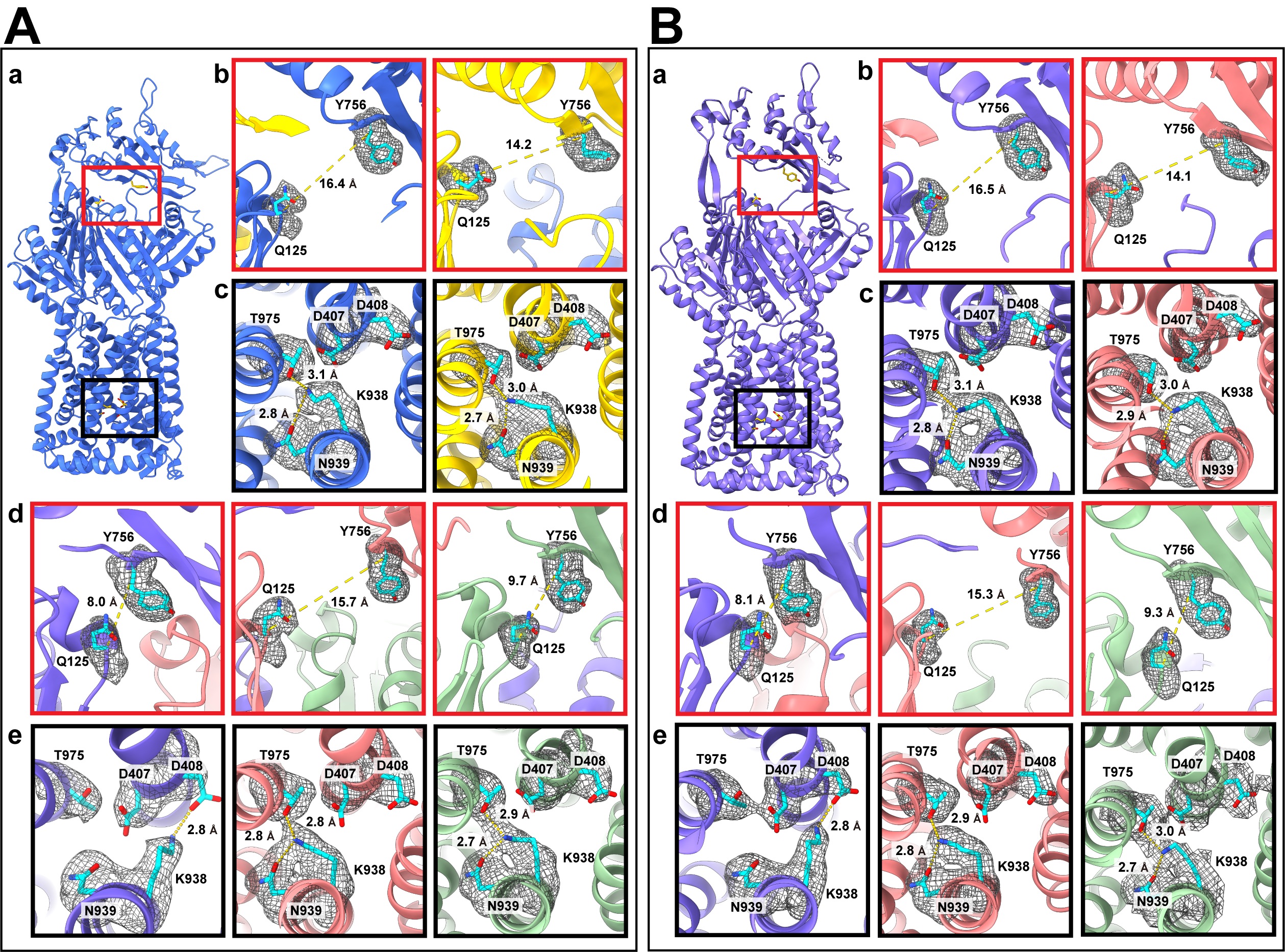

Supplement: FIG S2 [file mbio.03383-22-s0002.jpg]

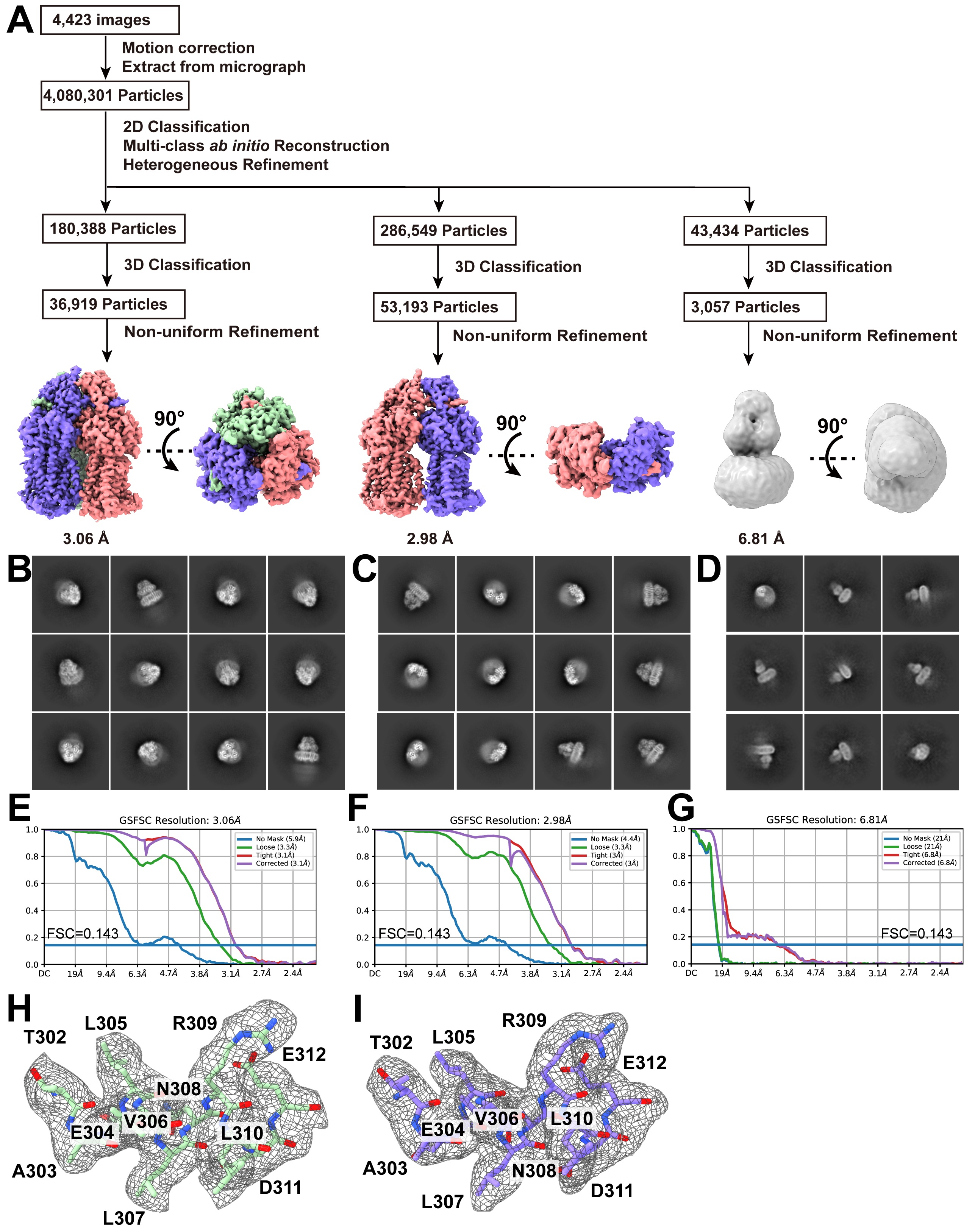

Supplement: FIG S3 [file mbio.03383-22-s0003.jpg]

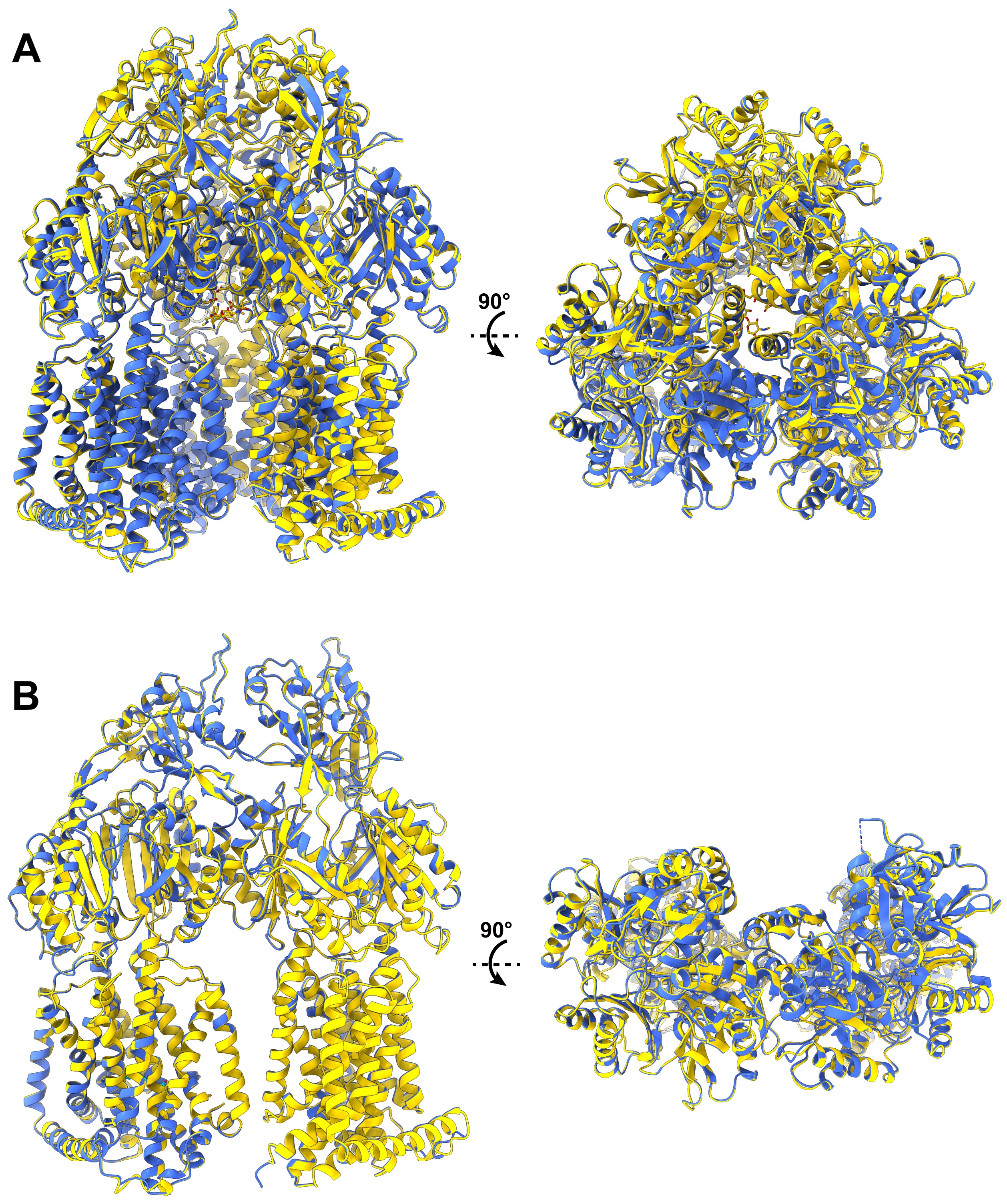

Supplement: FIG S4 [file mbio.03383-22-s0004.jpg]

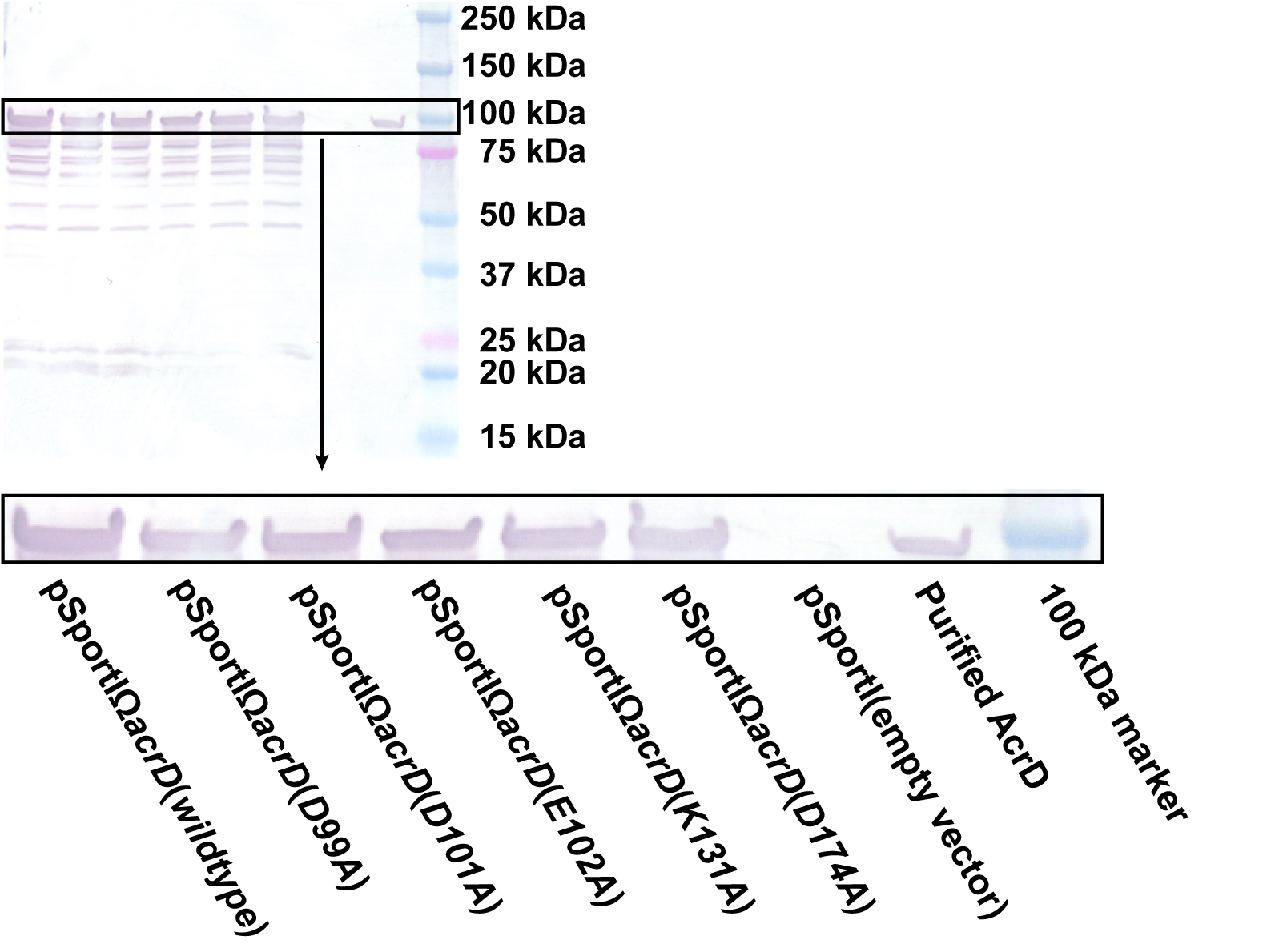

Supplement: FIG S5 [file mbio.03383-22-s0005.jpg]

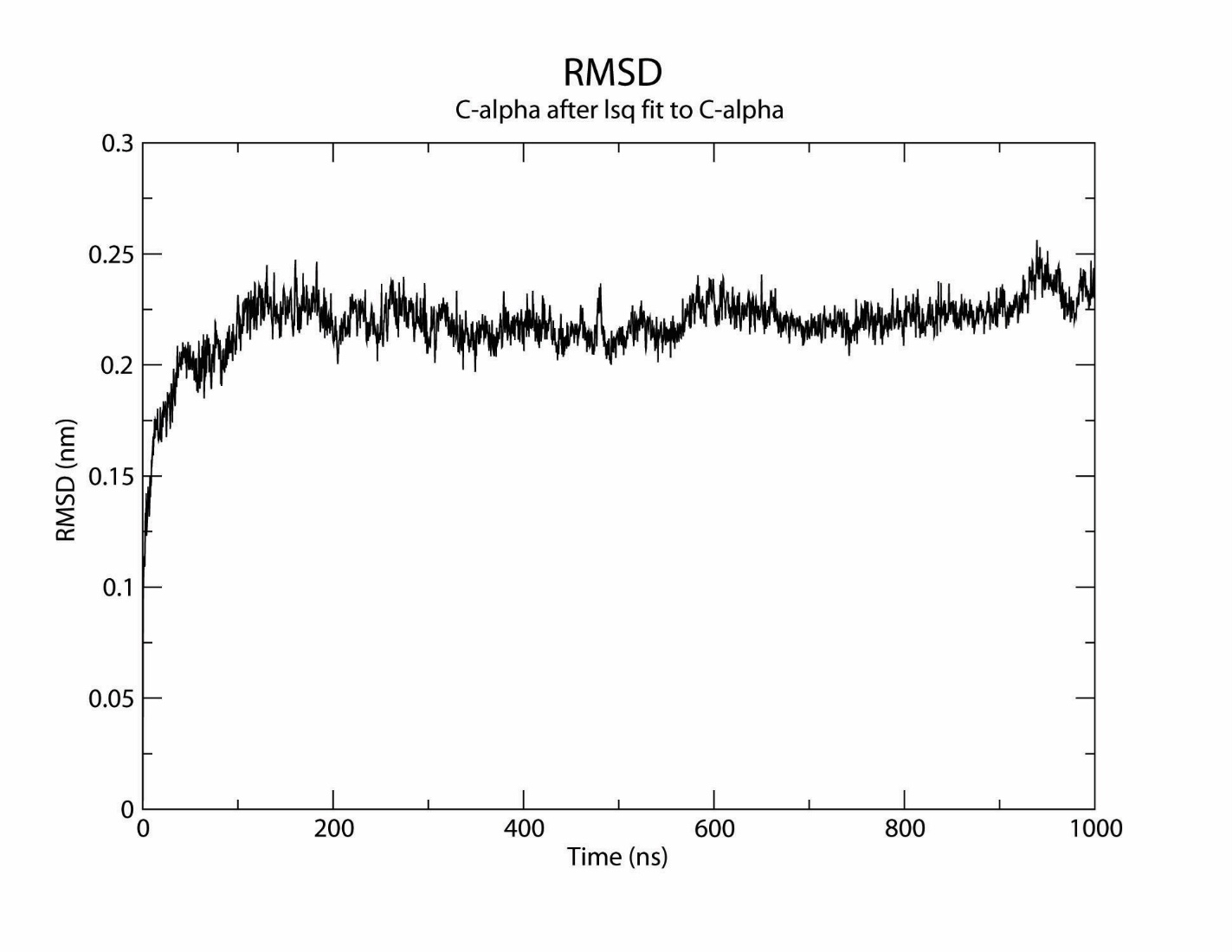

Supplement: FIG S6 [file mbio.03383-22-s0006.jpg]

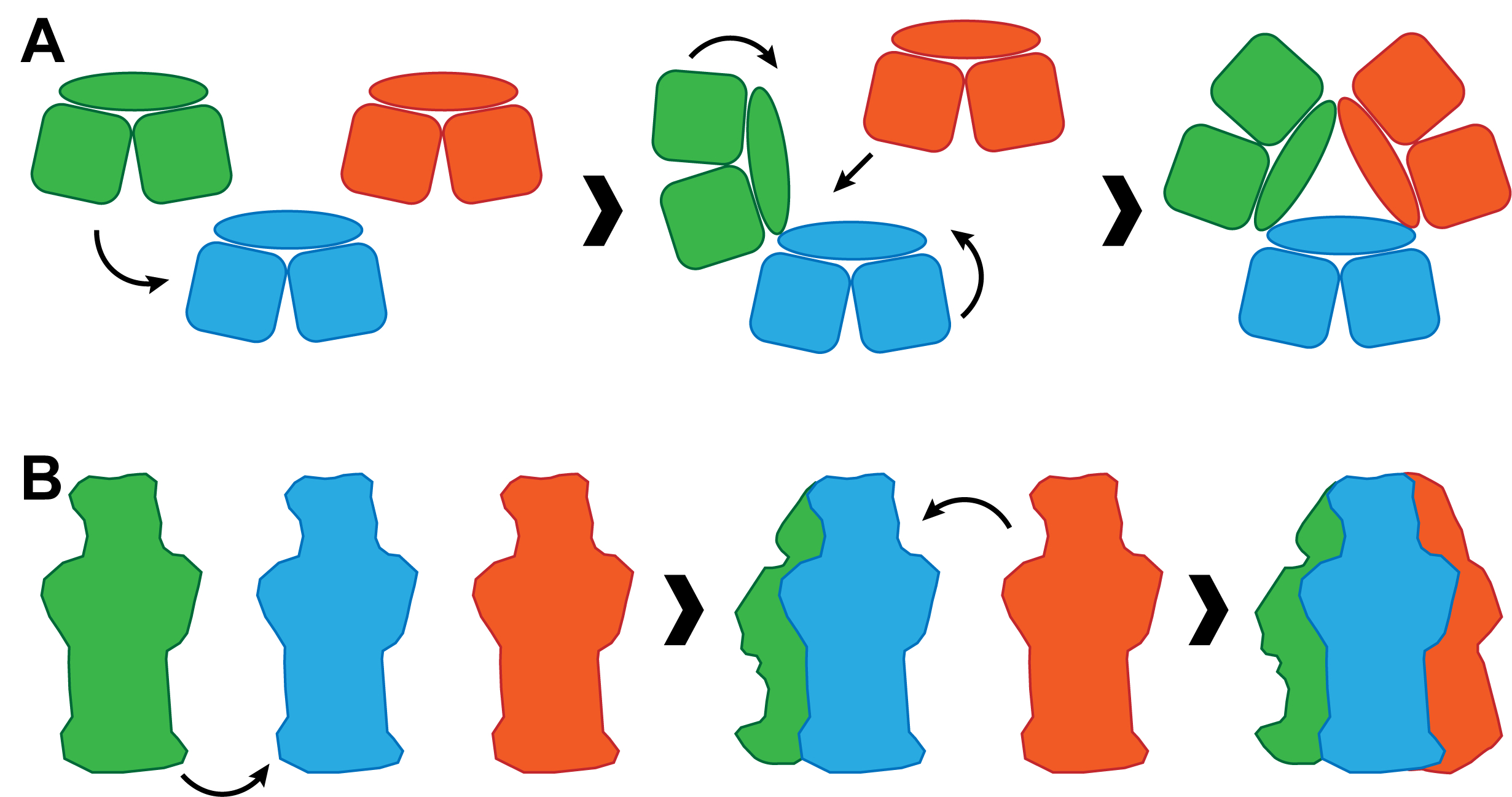

Supplement: FIG S7 [file mbio.03383-22-s0007.jpg]

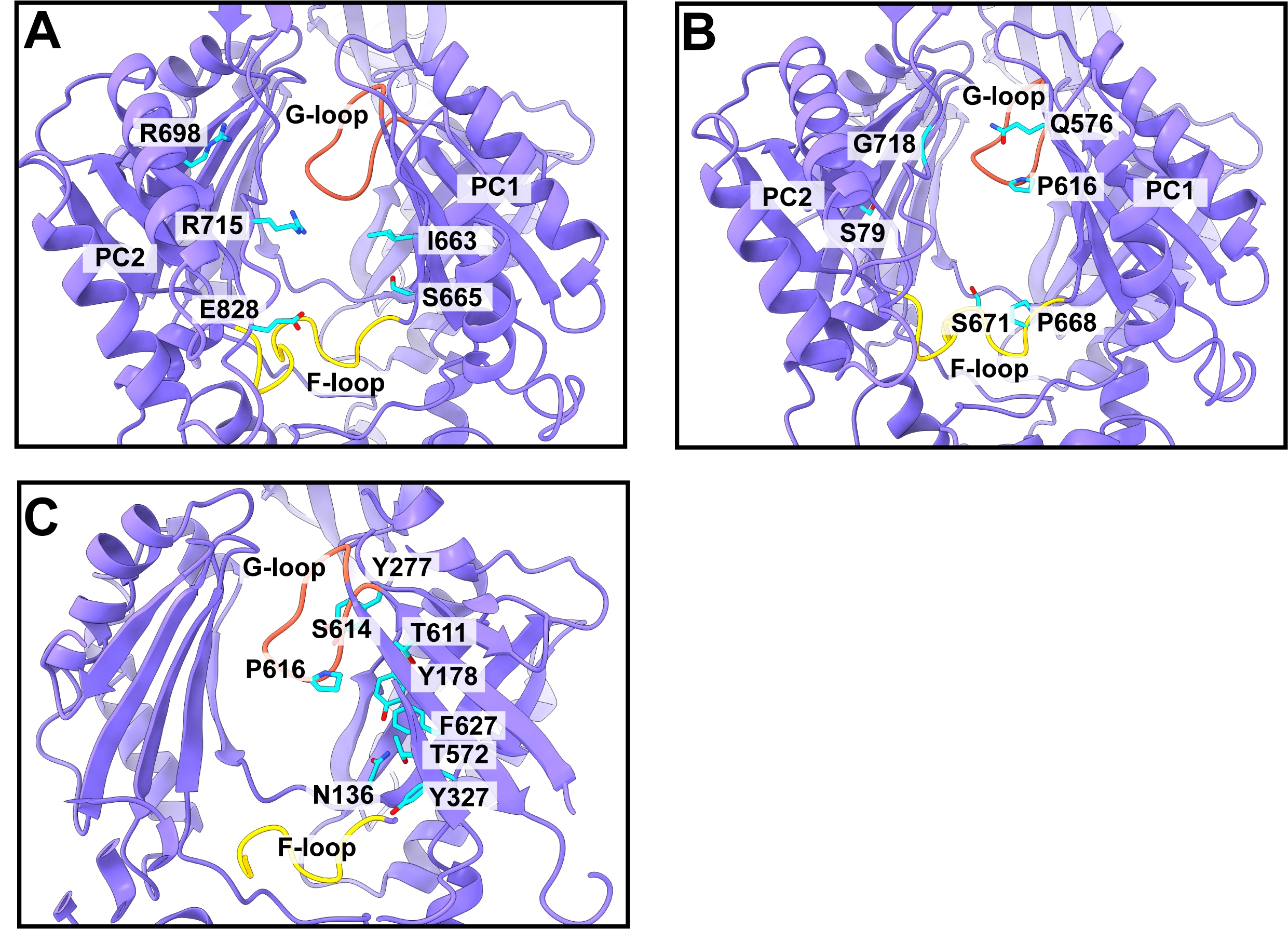

Supplement: FIG S8 [file mbio.03383-22-s0008.jpg]
