# Supplementary material for: Cryo-EM Structures of AcrD Illuminate a Mechanism for Capturing Aminoglycosides from Its Central Cavity
Source: mBio. 2023 Jan 10;14(1):e03383-22. doi: 10.1128/mbio.03383-22 (PMC9973356; doi:10.1128/mbio.03383-22)
Supplement: TABLE S1 [file mbio.03383-22-s0009.pdf]

Table S1. AcrD cryo-EM data collection and refinement statistics.

**Data collection**	**AcrD in the absence of gentamicin**			**AcrD in the presence of gentamicin**	
Magnification	81,000	81,000	81,000	81,000	81,000
Voltage (kV)	300	300	300	300	300
Electron Microscope	Krios-GIF-K3	Krios-GIF-K3	Krios-GIF-K3	Krios-GIF-K3	Krios-GIF-K3
Defocus (um)	-0.8 to -1.5	-0.8 to -1.5	-0.8 to -1.5	-0.8 to -1.5	-0.8 to -1.5
Energy filter width (eV)	20	20	20	20	20
Pixel size (Å)	1.07 (0.535)	1.07 (0.535)	1.07 (0.535)	1.07 (0.535)	1.07 (0.535)
Total dose ($e^-/Å^2$)	35.7	35.4	35.7	35.9	33
Number of frames	39	39	39	38	40
Number of micrographs	3,096	1,331	2,593	1,990	2,433
Number of Initial particles	7,192,708			4,080,301	
**Refinement**	**Trimeric AcrD**		**Dimeric AcrD**	**Trimeric AcrD**	**Dimeric AcrD**
Number of total particles	99,954		182,130	36,919	53,193
GS-FSC Resolution (0.143, Å)[a]	3.09		2.95	3.06	2.98
Model composition					
Chains	3		2	3	2
Protein residues	3,095		2,043	3,097	2,039
Ligand	0		0	1	0
r.m.s.d.					
Bond lengths (Å)	0.003		0.003	0.004	0.004
Bond angles (°)	0.504		0.499	0.524	0.513
**Validation**					
MolProbity score	1.74		1.67	1.78	1.82
Clash score	7.00		7.44	7.15	8.70
Ramachandran plot					
Favored (%)	98.06		98.33	98.87	97.88
Allowed (%)	1.94		1.67	1.10	2.12
Disallowed (%)	0.00		0.00	0.03	0.00
CC Mask	0.72		0.75	0.77	0.74

[a]Gold-Standard Fourier-Shell Correlation
