# Supplementary material for: Cryo-EM Structures of AcrD Illuminate a Mechanism for Capturing Aminoglycosides from Its Central Cavity
Source: mBio. 2023 Jan 10;14(1):e03383-22. doi: 10.1128/mbio.03383-22 (PMC9973356; doi:10.1128/mbio.03383-22)
Supplement: TABLE S2 [file mbio.03383-22-s0010.pdf]

Table S2. State assignment of AcrD protomer.

Protomer	Cleft State	Exit site distance, Q125 to Y756	Hydrogen-bonded distance, K938 to				Protomer Assignment
			D407 (Å)	D408 (Å)	N939 (Å)	T975 (Å)	
**In the absence of gentamicin**							
Trimeric AcrD, A	Open	8.0	-	2.8	-	-	Binding
Trimeric AcrD, B	Closed	15.7	-	-	2.8	2.8	Extrusion
Trimeric AcrD, C	Closed	9.7	-	-	2.7	2.9	Resting
Dimeric AcrD, A	Closed	16.4	-	-	2.8	3.1	Extrusion
Dimeric AcrD, B	Closed	14.2	-	-	2.7	3.0	Extrusion
**In the presence of gentamicin**							
Trimeric AcrD, A	Open	8.1	-	2.8	-	-	Binding
Trimeric AcrD, B	Closed	15.3	-	-	2.8	2.9	Extrusion
Trimeric AcrD, C	Closed	9.3	-	-	2.7	3.0	Resting
Dimeric AcrD, A	Closed	16.5	-	-	2.8	3.1	Extrusion
Dimeric AcrD, B	Closed	14.1	-	-	2.9	3.0	Extrusion

Protomer states were defined using three criteria. (1) State of the periplasmic cleft between PC1 and PC2. (2) Size of the extrusion tunnel measured by the distance between the Cα atoms of Q125 and Y756, which form the substrate exit site. (3) Hydrogen-bonded distance between the "proton sweeper" K938 and other residues within the proton-relay network (D407, D408, N939 or T975). Only distances of 3.20 Å and under are noted.
